# Selenium and other heavy metal levels in different rice brands commonly consumed in Pretoria, South Africa

**DOI:** 10.1016/j.heliyon.2024.e29757

**Published:** 2024-04-22

**Authors:** Oluwaseun Mary Oladeji, Kgomotso Magoro, Liziwe Lizbeth Mugivhisa, Joshua Oluwole Olowoyo

**Affiliations:** a Department of Biology and Environmental Science, Sefako Makgatho Health Sciences University, Pretoria, South Africa, P.O. Box 139, 0204; b Department of Health Science and The Water School, Florida Gulf Coast University, Fort Myers, USA

**Keywords:** Selenium, ICP-MS, Heavy metals, Rice, Pretoria, South Africa

## Abstract

For centuries, rice has been a dietary staple food partially due to its accessibility, affordability, and nutritional content. However, it has been documented that plants can bioaccumulate trace elements from soil and store them in their tissues therefore necessitating monitoring of its nutritional quality. The current study investigated the Selenium and heavy metal contents of various brands of rice obtained from different retail stores in Pretoria, South Africa. The analysis was carried out using different rice samples and different methods/stages of cooking rice including the analysis of rinsed rice water (RW), raw rice (RR), cooked rice (CR), and cooked rice water (CW), for trace elements content using the Inductive Couple Plasma Mass Spectrometry. The results revealed that the Se content ranged from 0.013 ± 0.01 mg/kg - 0.089 ± 0.06 mg/kg in RR, 0.013 ± 0.01 mg/kg - 0.046 ± 0.01 mg/kg in CR, 0.01 ± 0.01mg/kg– 0.028 ± 0.00 mg/kg in RW and 0.01 ± 0.01 mg/kg - 0.048 ± 0.01 mg/kg in CW. The calculated estimated dietary intake (EDI) of Se was recorded as follows; raw rice (7.06 × 10−5 mg/day), cooked rice (5.01 × 10^−5^ mg/day), water from cooked rice (4.54 × 10^−5^ mg/day) and rinsed water of raw rice (3.97 × 10^−5^ mg/day). The concentrations of all other heavy metals measured were within the WHO-recommended limits. The HQ for all the trace metals in all the samples did not exceed one, implying that there is no health risk from trace metals analysed in this study from the consumption of the rice brands used in this study. The results of this study demonstrated that reliance on rice alone for the supply of Se may be inadequate owing to the values obtained in our study. Constant monitoring of the nutritional contents of food products may be required to improve the overall nutritional well-being of the consumers.

## Highlights

• Rice has been a dietary staple food.
• The concentrations of selenium and other heavy metals were reported in different rice extracts.
• The Hazard Quotient (HQ) for all the heavy metals in all the samples did not exceed one.
• There are no health risk associated with the intake of the different rice extracts used in the study.

## 1 Introduction

Rice (*Oryza sativa* L.) is the main food for more than a billion people around the world and a significant source of energy in foods [1]. Rice is selected by 70 % of the population as the main staple food and it contributes more than 60 % of the daily dietary selenium intake [2]. Asia is the world's largest producer of rice grains, accounting for roughly 90 % of total production, followed by South America [3,4]. Rice requires an enormous amount of water to grow, and this water may be contaminated with different types of pollutants from both natural and man-made sources throughout the farming system [5].

Food is a complex mixture of different ingredients that are divided into nutrients and non-nutrients. It has long been accepted that nutrients are food, giving cells the building blocks they require to grow and proliferate as well as the energy to drive cellular metabolism [6]. To maintain healthy growth in humans, different nutritional qualities must be met and adequate [7]. In general, the consumption of food is expected to provide these nutrients in humans and rice for instance is believed to increase the levels of Se in humans if adequate quantities are contained in the rice [8].

Selenium (Se) is an important micronutrient for human and animal health. Several studies have revealed that it can scavenge free radicals, coordinate immune responses, and delay immune system aging [9,10]. In 1973, the World Health Organization declared Se to be a necessary trace element for humans [11], and this was confirmed in 1988 when the Chinese Nutrition Society revised dietary nutrients, listing Se as one of the 15 daily essential dietary nutrients [12]. Extensive studies have been conducted to understand the complex relationship between Se-containing chemicals and human health, as well as the dietary requirements for Se and its amounts in different foods [11]. Even though the amount of Se required for human consumption is extremely low, Se is relatively rare in the Earth's crust. Its insufficiency has been identified as a global human health issue that requires immediate attention [13].

However, during rice cultivation using various methods, the rice can bio-accumulate pollutants such as heavy metals from the soil thereby limiting its role in supplying essential nutrients for humans. The study of TatahMentan et al. [14] showed that rice may contain toxic heavy metals which are not required in humans. The rising public health risks connected with consuming heavy metals from crops in our diets are a significant source of worry. Heavy metals are persistent and non-biodegradable elements [15], such that they might build up in plant tissues, invade the food chain, and ultimately build up in major human organs [16]. In terms of human health, heavy metals can be detrimental [17]. They can accumulate in plant tissues and be released under specific physicochemical conditions, entering the food chain and eventually accumulating in essential human organs following ingestion [18].

Cadmium (Cd), mercury (Hg), arsenic (As), and lead (Pb) are among the most toxic heavy metals to humans, plants, and soil microbial populations [19]. These metals' negative effects on human life are caused by bioaccumulation and bio-augmentation in the food chain [20]. The growth media, soil, fertilizers, pesticides, vehicle exhausts, and nutrient solutions are the primary sources of heavy metals in plants [21]. Heavy metal consumption causes a variety of disorders because heavy metals are mutagenic, teratogenic, and carcinogenic [22]. Heavy metals can cause irreversible brain damage, cardiovascular illnesses, cirrhosis, dementia, bleeding, encephalopathy, renal dysfunction, bone disorders, impaired sperm motility, and gastrointestinal malignancies in humans [23,24].

The Se content of selenium-rich rice available in the market varies due to differences in varieties, soil environmental conditions, sources of Se [25,26], and Se application methods. Therefore, the Se content of selenium-rich rice on the market may vary, making it difficult to accurately ascertain the population's dietary selenium intake. Moreover, due to the heat effects on rice during cooking and the washing process, Se levels in rice might be lost resulting in a deficiency in consumers [27]. To the best of our knowledge, evaluation of some food items for their nutritional content including rice for Se content in Africa and in this instance in South Africa has not been carried out and where available the information is still very scanty. In addition, most of the studies focused on the Se content of selenium-rich in raw rice, while there are few reports on cooked rice, rinsed water, water from cooked rice, and their health risk because of insufficient or excessive Se intake. The current study therefore conducted an in-depth analysis on the Se content and heavy metal contents of different rice brands purchased from different stores in Pretoria. The study further determined the differences in the Se content of both cooked and uncooked rice and estimated rice Se intake with its associated risks in Pretoria, South Africa.

## 2 Materials and methods

### 2.1 Sampling collection

A total of 10 different brands of rice commonly consumed in Pretoria, South Africa were purchased from different shops. The sampling was done around June 2022.

### 2.2 Sample extraction

#### 2.2.1 Sample preparation: digestion process for raw rice, cooked rice, rinsed rice water before cooked and water from cooked rice

Ten different pots were used in this experiment. Each pot was rinsed with distilled water to prevent cross-contamination following Welna et al. [28] with little modification. A quantity of 150g of the raw rice was placed in the pots, rinsed and the rinsing water was collected and placed into 2L bottles followed by cooking of the rinsed rice. Another set of raw rice samples was used (150g) for the analysis. The grains of rice (i.e. cooked rice and uncooked rice) were dried at 60 °C to a constant weight [29], and then crushed to powder with a blender. The dried powders of all samples (0.5 g) were subjected to acid digestion to analyse the metals with the use of ICP-MS.

#### 2.2.2 Acid digestion

All samples were digested using 10 mL HNO_3_:HClO_4_ (9:1) in a 100 mL beaker. The digestion solution was heated at 60 °C and kept there for at least 3 h until it became colorless. After cooling, the digest was allowed to cool and then filtered using 0.45-μm pore size Whatman filter papers and then toped up to 50 mL with deionized water. Then the digests were transferred to a 50 mL capacity bottle. The Se and metals concentrations in the digested solutions were determined using inductively coupled plasma mass spectrometry (ICP-MS) [30,31].

### 2.3 Health risk assessment for humans and estimated dietary intake calculation

The daily intake of Se in rice was calculated using equation (1). The estimated daily intake (EDI) of Se in the four rice extracts were calculated using the equation described below [32].

(1) DI=D(g)×Cc(mg/kg)

Where DI is the daily intake,

D(g) is the average daily intake of rice (in g/day),

and *Cc* is the calculated concentration of the elements (μg/g).

EDI were expressed in mg/kg BW/day was calculated as in the following equation (2):

(2) $$EDI=\frac{\frac{DI}{BW}}{1000}$$

Where BW is the average body weight (65 kg).

Hazard quotients (HQ) for Se were calculated in all samples using the following equation 3

(3) $$HQ=\frac{EDI}{RFD}$$

Where RfD - 0.005 mg/kg/day for Se.

If the HQ value was less than one, the non-carcinogenic risk was assumed to be acceptable. HQ exceeds one, serious health impacts are possible [33].

#### 2.3.1 Statistical analysis

One-way analysis of variance (ANOVA) was carried out using the SPSS program version 28.0.

## 3 Result and discussion

**Fig. 1:**
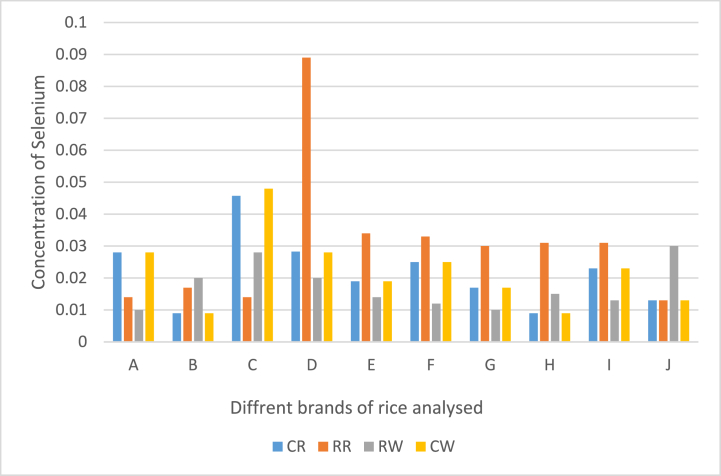
The concentration of selenium in raw rice (RR), rinsed water before cooked (RW), cooked rice (CR) and water from the cooked rice (CW).

In the present study the concentrations of Se and other heavy metals such as As, Cr, Cu, Pb, Mn, Fe, Mg, Mo, Ni, and Zn in all rice samples and water samples Table 1A, Table 1BA and 1Bfrom the rice are presented either in Fig. 1 and Table 1A, Table 1B below. Se concentrations in the different rice extracts range from 0.013 ± 0.01 mg/kg - 0.089 ± 0.06 mg/kg in raw rice, 0.013 ± 0.01–0.046 ± 0.01 mg/kg in cooked rice, 0.01 ± 0.00 mg/kg – 0.028 ± 0.00 mg/kg in rinsed raw rice water and 0.01 ± 0.00–0.048 ± 0.01 mg/kg in water from cooked rice. The highest concentration was found in the following order raw rice (RR), water from the cooked rice (CW), cooked rice (CR), and Rinse rice water (RW). The concentrations of Se in raw rice were higher than those recorded for cooked rice and water from the cooked rice, even though there are differences in the values obtained for Se in the cooked rice, raw rice, water from cooked rice, and rinsed water, the differences obtained were not significant statistically (P > 0.05). The effect of cooking or washing does not necessarily lead to changes in the values of Se. The present results were compared with the data that were previously reported in Saudi Arabia where values obtained for Se in rice ranged from (0.07–0.24 μg/g) [34] China (0.81–7.26 μg/g) [25] which also had lower Se content compared with our present result. When compared with other studies from, Egypt (0.15 μg/g) [35], Saudi Arabia (0.072 μg/g) [36], in Brazil (0.13 μg/g) [37], the results of the current study showed lower levels of Se. The decrease in Se content may also be due to volatilization or inadequate levels of Se in soils and prevents it from entering the harvested rice, increasing Se shortage in particular areas [38] and, to a lesser extent, the shoots, plants can volatilize selenium as dimethylselenide (DMSe) or dimethyldiselenide (DMDSe) [39]. This indicates that Se is more likely to be lost through heating processes of grains than other minerals [40,41]. This low level of Se may also contribute to the consumers' insufficient intake of Se if they rely solely on rice to supply the required Se in humans, which can compromise the immune system of the body, the thyroid, and cognitive development and increase the risk of non-communicable diseases [42]. Literature has suggested that the disparity in the concentrations of Se in plants across different geographical areas is chiefly a result of the variation of the total amount of selenium in the soil, but it is also influenced by the composition and pH of the soil environment [43]. The content of Se in the soil has a major impact on the levels of Se in plants. This assertion is supported by the reduced uptake of essential elements, seed germination and growth being hindered, suppressed photosynthesis and oxidative stress and cell membrane damage occurring [44,45]. Though Se plays an essential role in mitigating the toxicity caused by heavy metals and decreasing their build-up in plants [46], anthropogenic processes, such as mineral smelting and processing, as well as coal burning, can result in higher levels of Se in the soil, thus providing plants with a beneficial supplement as Se is usually scarce in most soils [47].

**Table 1A:** Concentration of other heavy metals analysed (mg/kg) in raw rice (RR) and cooked rice (CR).

Sample A (RW and CR)	As	Cr	Cu	Fe	Mg	Mn	Mo	Ni	Pb	Zn
CR A	0.013 ± 0.14	0.055 ± 0.004	0.060 ± 0.010	1.651 ± 0.076	25.550 ± 1.366	0.133 ± 0,001	0.107 ± 0,005	0.017 ± 0,015	ND	0.219 ± 0.018
RR A	ND	0.034 ± 0.009	0.034 ± 0.005	0.876 ± 0.038	1.574 ± 0.052	0.063 ± 0.002	0.015 ± 0.009	0.015 ± 0.014	ND	0.129 ± 0.002
CR B	0.025 ± 0.005	0.038 ± 0.005	0.091 ± 0.013	0.743 ± 0.031	29.583 ± 2.482	1.472 ± 0.001	0.044 ± 0.006	0.014 ± 0.013	ND	0.204 ± 0.022
RR B	ND	0.035 ± 0.009	0.039 ± 0.006	0.693 ± 0.032	4.191 ± 0.240	0.050 ± 0.001	0.013 ± 0.005	0.009 ± 0.001	ND	0.118 ± 0.003
CR C	0.007 ± 0.012	0.047 ± 0.006	0.10 ± 0.014	0.966 ± 0.040	34.002 ± 2.697	0.133 ± 0.000	0.084 ± 0.009	0.022 ± 0.020	ND	0.246 ± 0.025
RR C	ND	0.171 ± 0.009	0.038 ± 0.006	1.168 ± 0.056	2.408 ± 0.154	0.051 ± 0.002	0.020 ± 0.013	0.046 ± 0.041	ND	0.110 ± 0.002
CR D	0.003 ± 0.005	0.041 ± 0.006	0.038 ± 0.011	0.756 ± 0.033	32.122 ± 2.727	0.08 ± 0.0004	0.036 ± 0.006	0.014 ± 0.013	ND	0.373 ± 0.027
RR D	ND	0.713 ± 0.030	0.054 ± 0.007	3.198 ± 0.177	4.071 ± 0.211	0.107 ± 0.002	0.031 ± 0.013	0.181 ± 0.084	ND	0.164 ± 0.006
CR E	0.001 ± 0.002	0.043 ± 0.006	0.053 ± 0.012	0.889 ± 0.039	31.286 ± 2.247	0.163 ± 0.000	0.061 ± 0.009	0.029 ± 0.025	ND	0.185 ± 0.022
RR E	ND	0.035 ± 0.011	0.031 ± 0.006	0.629 ± 0.030	3.348 ± 0.146	0.045 ± 0.001	0.014 ± 0.009	0.011 ± 0.011	ND	0.092 ± 0.002
CR F	0.01 ± 0.017	0.044 ± 0.006	0.099 ± 0.015	0.91 ± 0.041	36.317 ± 2.529	0.108 ± 0.000	0.080 ± 0.001	0.02 ± 0.018	ND	0.237 ± 0.023
RR F	ND	0.037 ± 0.011	0.041 ± 0.007	0.525 ± 0.024	4.066 ± 0.224	0.045 ± 0.001	0.016 ± 0.013	0.010 ± 0.01	ND	0.098 ± 0.003
CR I	ND	0.041 ± 0.005	0.082 ± 0.012	1.016 ± 0.047	23.915 ± 1.019	0.127 ± 0.001	0.137 ± 0.01	0.019 ± 0.028	ND	0.304 ± 0.016
RR G	ND	0.092 ± 0.011	0.040 ± 0.008	0.700 ± 0.036	7.110 ± 0.285	0.281 ± 0.001	0.016 ± 0.012	0.024 ± 0.022	ND	0.141 ± 0.006
CR H	0.004 ± 0.007	0.088 ± 0.007	0.15 ± 0.017	1.651 ± 0.087	50.135 ± 6.315	0.661 ± 0.002	0.05 ± 0.007	0.028 ± 0.024	ND	0.493 ± 0.041
RR H	ND	0.035 ± 0.012	0.037 ± 0.006	0.892 ± 0.045	2.560 ± 0.32	0.043 ± 0.001	0.014 ± 0.011	0.010 ± 0.011	ND	0.180 ± 0.004
CR I	0.010 ± 0.018	0.033 ± 0.007	0.081 ± 0.012	0.779 ± 0.038	20.751 ± 0.716	0.204 ± 0.001	0.101 ± 0.011	0.011 ± 0.01	ND	0.555 ± 0.019
RR I	ND	0.035 ± 0.011	0.058 ± 0.008	0.717 ± 0.035	4.225 ± 0.215	0.040 ± 0.001	0.019 ± 0.011	0.024 ± 0.022	ND	0.111 ± 0.004
CR J	0.01 ± 0.011	0.054 ± 0.008	0.082 ± 0.013	1.05 ± 0.054	34.926 ± 2.964	0.140 ± 0.001	0.142 ± 0.008	0.043 ± 0.037	ND	0.649 ± 0.033
RR J	ND	0.056 ± 0.001	0.035 ± 0.011	0.082 ± 0.013	3.050 ± 0.154	0.140 ± 0.001	0.049 ± 0.001	0.007 ± 0.011	ND	0.110 ± 0.004

Cooked rice denote CR, Raw rice denote RR.

**Table 1B:** Concentration of other heavy metals analysed (mg/kg) in rinsed water before cooked (RW) and water from cooked rice (CW).

Sample B (RW and CW	As	Cr	Cu	Fe	Mg	Mn	Mo	Ni	Pb	Zn
RW A	0.007 ± 0.007	0.035 ± 0.005	0.042 ± 0.006	1.415 ± 0.061	7.471 ± 0.208	0.143 ± 0.001	0.052 ± 0.000.	0.0104 ± 0.010	ND	0.123 ± 0.007
CW A	0.013 ± 0.014	0.055 ± 0.004	0.060 ± 0.010	1.651 ± 0.076	25.550 ± 1.366	0.133 ± 0.001	1.07 ± 0.005	0.017 ± 0,015	ND	0.219 ± 0.018
RW B	0.01 ± 0.018	0.074 ± 0.006	0.099 ± 0.014	1.298 ± 0.057	36.689 ± 3.198	0.100 ± 0.001	0.120 ± 0.009	0.023 ± 0.021	ND	0.246 ± 0.025
CW B	0.025 ± 0.005	0.038 ± 0.005	0.091 ± 0.013	0.743 ± 0.031	29.583 ± 2.48	1.472 ± 0.001	0.044 ± 0.006	0.014 ± 0.013	ND	0.204 ± 0.022
RW C	0.006 ± 0.007	0.047 ± 0.007	0.116 ± 0.017	1.089 ± 0.051	50.163 ± 6.579	0.480 ± 0.001	0.041 ± 0.008	0.043 ± 0.038	ND	0.311 ± 0.040
CW C	0.007 ± 0.012	0.047 ± 0.006	0.10 ± 0.014	0.966 ± 0.040	34.002 ± 2.697	0.133 ± 0.000	0.084 ± 0.009	0.022 ± 0.02	ND	0.246 ± 0.025
RW D	0.007 ± 0.010	0.059 ± 0.008	0.113 ± 0.017	1.374 ± 0.066	64.53 ± 10.851	0.384 ± 0.001	0.028 ± 0.007	0.019 ± 0.017	ND	0.897 ± 0.069
CW D	0.003 ± 0.005	0.041 ± 0.006	0.038 ± 0.011	0.756 ± 0.033	32.12 ± 2.727	0.089 ± 0.000	0.036 ± 0.006	0.014 ± 0.013	ND	0.373 ± 0.027
RW E	0.007 ± 0.013	0.064 ± 0.007	0.118 ± 0.017	2.412 ± 0.117	60.990 ± 9.597	0.815 ± 0.001	0.047 ± 0.008	0.030 ± 0.027	ND	0.555 ± 0.053
CW E	0.001 ± 0.002	0.043 ± 0.01	0.053.±0.012	0.889 ± 0.039	31.286 ± 2.247	0.163 ± 0.000	0.061 ± 0.009	0.029 ± 0.025	ND	0.185 ± 0.022
RW F	0.004 ± 0.006	0.060 ± 0.006	0.142 ± 0.017	1.146 ± 0.056	47.618 ± 5.802	0.449 ± 0.001	0.043 ± 0.008	0.017 ± 0.025	ND	0.809 ± 0.046
CW F	0.01 ± 0.00	0.044 ± 0.005	0.099 ± 0.015	0.91 ± 0.041	36.317 ± 2.529	0.108 ± 0.000	0.080 ± 0.009	0.02 ± 0.018	ND	0.237 ± 0.023
RW G	0.002 ± 0.003	0.033 ± 0.007	0.121 ± 0.013	0.954 ± 0.047	27.114 ± 1.731	0.197 ± 0.001	0.034 ± 0.006	0.040 ± 0.057	ND	0.291 ± 0.019
CW G	ND	0.041 ± 0.005	0.082 ± 0.012	1.016 ± 0.047	23.915 ± 1.019	0.127 ± 0.001	0.137 ± 0.01	0.019 ± 0.028	ND	0.304 ± 0.016
RW H	0.006 ± 0.01	0.054 ± 0.008	0.171 ± 0.021	1.219 ± 0.057	70.77 ± 12.768	1.066 ± 0.001	0.052 ± 0.007	0.762 ± 0.065	ND	0.762 ± 0.065
CWH	0.004 ± 0.007	0.088 ± 0.007	0.15 ± 0.017	1.651 ± 0.087	50.14 ± 6.32	0.661 ± 0.00	0.05 ± 0.007	0.028 ± 0.024	ND	0.493 ± 0.041
RW I	0.003 ± 0.005	0.059 ± 0.008	0.106 ± 0.015	1.591 ± 0.082	49.698 ± 6.034	0.435 ± 0.001	0.032 ± 0,008	0.017 ± 0.025	ND	0.476 ± 0.040
CW I	0.010 ± 0.018	0.033 ± 0.007	0.081 ± 0.012	0.779 ± 0.038	20.751 ± 0.716	0.204 ± 0.00	0.101 ± 0.011	0.011 ± 0.01	ND	0.555 ± 0.019
RW J	0.007 ± 0.012	0.040 ± 0.007	0.049 ± 0.011	0.773 ± 0.034	24.360 ± 0.785	0.267 ± 0.001	0.062 ± 0.011	0.013 ± 0.012	ND	0.402 ± 0.018
CW J	0.01 ± 0.011	0.054 ± 0.008	0.082 ± 0.013	1.05 ± 0.054	34.926 ± 2.96	0.140 ± 0.00	0.142 ± 0.008	0.043 ± 0.037	ND	0.649 ± 0.033

Rinsed water before cooked denote RW and water from the cooked rice denote CW.

Other heavy metals, including Fe, As, Cr, Cu, Ni, Mg, Mn, Mo, and Zn were detected in all the rice extracts as shown in Fig. 1 and. The following ranges were detected: Fe (0.743 ± 0.031 mg/kg – 2.412 ± 0.117 mg/kg), As (ND – 0.025 ± 0.005 mg/kg), Cr (ND–0.088 ± 0.007 mg/kg), Cu (0.031 ± 0.006 mg/kg – 0.171 ± 0.021 mg/kg), Ni (0.009 ± 0.001 mg/kg – 0.762 ± 0.065 mg/kg), Mg (1.574 ± 0.052 mg/kg – 70.77 ± 12.768 mg/kg), Mn (0.384 ± 0.001 mg/kg – 1.472 ± 0.001 mg/kg), Mo (0.013 ± 0.005 mg/kg −1.07 ± 0.005 mg/kg), Pb (ND) and Zn (0.092 ± 0.002 mg/kg – 0.809 ± 0.046 mg/kg). When compared with the permissible limits recommended for each metal, all the heavy metals analysed were below the limits recommended by WHO. Also, the current study was compared to other studies, and our results are similar [48,49].

One of the plants that has been demonstrated to absorb numerous hazardous metals from the soil is rice, which increases human exposure to metals everywhere, especially in Asian nations where 90 % of the world's production of rice is consumed [14]. Plant-derived foods that are grown on heavy metals polluted soils can easily absorb heavy metals from the soil [50], irrigation water [51], or heavy metal-contaminated sludge [52]. More effectively than other crops, rice may absorb harmful heavy metals from the soil and water in paddy fields through its root system [53,54]. It has been demonstrated that anaerobic soil conditions in paddy fields may cause hazardous metals to be mobilized more frequently and become more bioavailable to rice [55]. Alkaline soil (high pH) contains lower concentrations of Pb and other metals like Cr and Co reducing the danger of uptake through plants [56]. Heavy metals including Pb, Cd, Mn, and As can enter the body through ingestion and the gastrointestinal tract when food is consumed and cause serious health issues [57]. Additionally, breathing might allow heavy metals to enter the body.

### 3.1 Assessment of daily dietary selenium intake

#### 3.1.1 Daily intake (DI)

**Fig. 2:**
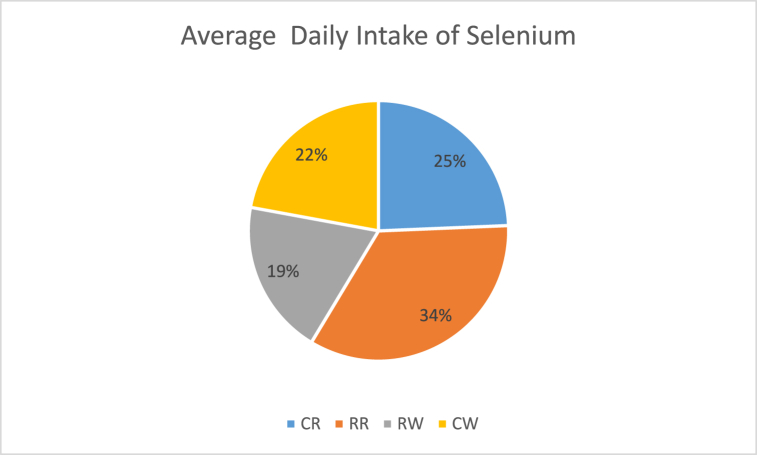
The variation of daily intake (DI) of Selenium in rice analysed.

The daily intake (mg/day) for Se in cooked rice (CR), raw rice (RR), rinse water (RW) and water from cooked rice (CW) were 1.35–6.86 mg/day, 2.10–13.35 mg/day, 1.50–4.20 mg/day and 1.35–7.20 mg/day respectively (Table 2). These results showed that the DI of Se in the rice analysed varied. The mean average daily intake was recorded in decreasing order RR > CR > CW > RW. These variations in the result could be due to differences in the samples. Regarding the typical daily intake, Fig. 2 shows the Se levels of each four extracts.

**Table 2:** Dietary intake and risk assessment of selenium in rice.

	CR (DI)	EDI	HQ	RR (DI)	EDI	HQ	RW (DI)	EDI	HQ	CW (DI)	EDI	HQ
**A**	4.20	6.46 × 10^−5^	1.3 × 10^−3^	2.1	3.23 × 10^−5^	6 × 10^−3^	1.5	2.31 × 10^−5^	5 × 10^−3^	1,5	2.31 × 10^−5^	4.62 × 10^−3^
**B**	1.35	2.08 × 10^−5^	4.0 × 10^−3^	2.5	3.92 × 10^−5^	8 × 10^−3^	3	4.62 × 10^−5^	9 × 10^−3^	1,35	2.08 × 10^−5^	4.15 × 10^−3^
**C**	6.86	1.05 × 10^−4^	2.11 × 0^−3^	2.1	3.23 × 10^−5^	6 × 10^−3^	4,2	6.46 × 10^−5^	1.3 × 10^−2^	7,2	1.11 × 10^−4^	2.22 × 10^−2^
**D**	4.25	6.53 × 10^−5^	1.3 × 10^−3^	13.3	2.05 × 10^−4^	4.1 × 10^−2^	3	4.62 × 10^−5^	9 × 10^−3^	4,2	6.46 × 10^−5^	1.29 × 10^−2^
**E**	2.85	4.38 × 10^−5^	9 × 10^−4^	5.1	7.85 × 10^−5^	1.6 × 10^−2^	2.1	3.23 × 10^−5^	6 × 10^−3^	2,85	4.38 × 10^−5^	8,77 × 10^−3^
**F**	3.75	5.77 × 10^−5^	1.2 × 10^−3^	4.95	7.62 × 10^−5^	1.5 × 10^−2^	1.8	2.77 × 10^−5^	6 × 10^−3^	3,75	5.77 × 10^−5^	1.15 × 10^−2^
**G**	2.55	3.92 × 10^−5^	8 × 10^−4^	4.5	6,92 × 10^−5^	1.4 × 10^−2^	1.5	2.31 × 10^−5^	5 × 10^−3^	2,55	3.92 × 10^−5^	7.85 × 10^−3^
**H**	1.35	2.08 × 10^−5^	4 × 10^−3^	4.65	7.15 × 10^−5^	1.4 × 10^−2^	225	3.46 × 10^−5^	7 × 10^−3^	1,35	2.08 × 10^−5^	4.15 × 10^−3^
**I**	3.45	5.31 × 10^−5^	1.1 × 10^−3^	4.65	7.15 × 10^−5^	1.4 × 10^−2^	1.95	3.00 × 10^−5^	6 × 10^−3^	3,45	5.31 × 10^−5^	1.06 × 10^−2^
**J**	1.95	3.00 × 10^−5^	6 × 10^−4^	1.95	3.00 × 10^−5^	6 × 10^−3^	4.5	6.92 × 10^−5^	1.4 × 10^−2^	1,35	2.08 × 10^−5^	4.15 × 10^−3^

#### 3.1.2 Estimated dietary intake (EDI) and hazard quotient of essential elements

Table 2 illustrated tthe he estimated dietary intake (EDI) of essential elements and the hazard quotient (HQ) for Se. The decreasing order of EDI was as follows: RR (7.06 × 10^−5^ mg/kg bw/day) > CR (5.01 × 10^−5^ mg/kg bw/day) > CW (4.54 × 10^−5^ mg/kg bw/day) > RW (3.97 × 10^−5^ mg/kg bw/day). The findings of this study showed the range of EDI (mg/kg bw/day) of the essential elements varies widely in the rice different extracts, RR, CR, RW, and the CW. Since our investigation was based on the intake from only four distinct extracts, a direct comparison cannot be done with the results in the literature. There are several reasons why the EDI between our study and those from Qatar, Italy, and Saudi Arabia differs (Se 9.55 × 10^−5^ – 5.75 × 10^−4^, Mn 1.49 × 10^−3^ – 3.31 × 10^−2^, Zn 1.33 × 10^−2^ – 5.83 × 10^−2^, Cu 1.65 × 10^−3^ – 5.42 × 10^−3^), (0.850–2.02), and 5.00 (3.17–7.65), 56.70 (36.08–8 6.70) and 66.53 (40.04–101.32) [34,58,59]. For instance, it is unlikely that different countries' soil quality, irrigation water consumption, and mineral content in animal feed will be comparable, which has an impact on how concentrated these components are in the main staple foods of these nations [60]. Other factors could be a result of environmental factors, such as cooking and washing, which are examples of environmental factors [61]. Moreover, our study focused on different extraction methods to figure out whether the four extracts examined in this study contained enough selenium to satisfy daily needs.

As shown in Table 2, the health risks were determined using the hazard quotient (HQ), which was computed using the EDIs and RFD of Se. The HQ for each of the Se in all the samples does not exceed one, due to the fact that their computed values for the HQ were less than 1.00, the findings for Se demonstrate that there is no health risk connected to consuming the rice used for the study for now.

## 4 Conclusion

The current study investigated the levels of Selenium and other heavy metals in some rice brands commonly consumed in some parts of South Africa. The study showed that all the heavy metals investigated in the study were all below the acceptable limit set by WHO, indicating that the different rice brands did not bioaccumulate heavy metals that may pose a threat to consumers at this stage. The levels of Se found in the rice brands are comparable to those that were previously reported by other researchers from different countries and may be increased to levels that are required for some of the rice brands. The different methods used in the preparation of rice to be consumed within the household did not significantly reduce the concentrations of Se present in the rice. However, since rice may be cultivated on different soil types which may then influence the levels of Se and other heavy metals in the rice, it will then be reasonable to monitor the levels of Se and other heavy metals periodically in other to ascertain their safety for human health. Further research may be required to investigate if the levels of Se found in the rice at this stage may be adequate as a nutrient for the consumers.

## Funding

This research received no external funding.

## Data availability statement

Data will be made available on request.

## CRediT authorship contribution statement

**Oluwaseun Mary Oladeji:** Writing – review & editing, Writing – original draft, Methodology, Investigation, Data curation, Conceptualization. **Kgomotso Magoro:** Methodology, Investigation, Data curation. **Liziwe Lizbeth Mugivhisa:** Writing – review & editing, Data curation, Conceptualization. **Joshua Oluwole Olowoyo:** Writing – review & editing, Writing – original draft, Supervision, Investigation, Funding acquisition, Conceptualization.

## Declaration of competing interest

The authors declare that they have no known competing financial interests or personal relationships that could have appeared to influence the work reported in this paper.
